# The selective disruption of presynaptic JNK2/STX1a interaction reduces NMDA receptor-dependent glutamate release

**DOI:** 10.1038/s41598-019-43709-2

**Published:** 2019-05-09

**Authors:** Serena Marcelli, Filomena Iannuzzi, Elena Ficulle, Dalila Mango, Stefano Pieraccini, Sara Pellegrino, Massimo Corbo, Maurizio Sironi, Anna Pittaluga, Robert Nisticò, Marco Feligioni

**Affiliations:** 1 Laboratory of Neuronal Cell Signaling, EBRI Rita Levi-Montalcini Foundation, Rome, 00161 Italy; 2 Laboratory of Neurobiology in Translational Medicine, Department of Neurorehabilitation Sciences, Casa Cura Policlinico, Milan, 20144 Italy; 3 Laboratory of Neuropharmacology, EBRI Rita Levi-Montalcini Foundation, Rome, 00161 Italy; 4 Department of Chemistry and National Inter-university Consortium for Materials Science and Technology -INSTM- UdR Milano, University of Milan, Milan, 20133 Italy; 5 Institute of Molecular Science and Technology, ISTM-CNR, Milan, 20133 Italy; 6 Department of Pharmaceutical Sciences, University of Milan, Milan, 20133 Italy; 7 Department of Pharmacy, Pharmacology and Toxicology Section, University of Genoa, Genoa, 16148 Italy; 8 Center of Excellence for Biomedical Research, University of Genoa, Genoa, 16132 Italy; 9 Department of Biology, University of Rome “Tor Vergata”, Rome, 00133 Italy

**Keywords:** Synaptic plasticity, Synaptic transmission

## Abstract

The neuronal loss caused by excessive glutamate release, or ‘excitotoxicity’, leads to several pathological conditions, including cerebral ischemia, epilepsy, and neurodegenerative diseases. Over-stimulation of presynaptic N-methyl-D-aspartate (NMDA) receptors is known to trigger and support glutamate spillover, while postsynaptic NMDA receptors are responsible for the subsequent apoptotic cascade. Almost all molecules developed so far are unable to selectively block presynaptic or postsynaptic NMDA receptors, therefore a deeper knowledge about intracellular NMDA pathways is required to design more specific inhibitors. Our previous work showed that presynaptic c-Jun N-terminal kinase 2 (JNK2) specifically regulates NMDA-evoked glutamate release and here we demonstrate that an interaction between Syntaxin-1a and JNK2 is fundamental to this mechanism. Based on this evidence, a new cell permeable peptide (CPP), “JGRi1”, has been developed to disrupt the JNK2/STX1a interaction to indirectly, but specifically, inhibit presynaptic NMDA receptor signaling. JGRi1 reduces the NMDA-evoked release of glutamate both in *in-vitro* and *ex-vivo* experiments while also being able to widely diffuse throughout brain tissue via intraperitoneal administration. In conclusion, the JNK2/STX1 interaction is involved in presynaptic NMDA-evoked glutamate release and the novel CPP, JGRi1, acts as a pharmacological tool that promotes neuroprotection.

## Introduction

The excess of glutamate release is recognized as a major cause for the massive neuronal death occurring in several pathologies including stroke, traumatic brain injury, spinal cord injury, epilepsy, and neurodegenerative conditions such as Amyotrophic Lateral Sclerosis (ALS), Alzheimer’s and Huntington’s diseases^1^. The neurotoxic activity of glutamatergic release is mainly expressed through the over-activation of *N*-methyl-D-aspartate (NMDA) glutamate receptors that, in turn, induce the rapid depolarization of the plasma membrane and an abrupt Ca^2+^ influx, ultimately resulting in further release of glutamate and neuronal death^2–4^. The pharmacological inhibition of NMDA receptors would be the elective treatment against ‘excitotoxicity’ but no such molecule has reached the market so far^5^. A possible explanation for the failure of the compound to reach the market could be toxicity resulting from the total, unselective blockade of synaptic transmission mediated by NMDA receptors^5^.

A more efficient and less toxic strategy would be to selectively block NMDA receptors by targeting the intracellular signalling pathways that mostly contribute to the excessive glutamate overflow. Recently, a promising approach using Tat-linked peptides was successful in reducing neuronal toxicity supported by disrupting the interaction of postsynaptic NMDA receptors with postsynaptic protein PSD-95^6–9^. Similarly, the highly conserved signalling pathway of mitogen-activated protein (MAP) kinases, including JNK proteins, has been identified as a postsynaptic pharmacological target downstream of NMDA receptor activation, especially during cerebral ischemia, epilepsy or hypoxic conditions^10–12^. Indeed, the inhibition of postsynaptic NMDA receptors signalling pathways is an interesting approach to reduce excitotoxicity but intervening at the presynaptic side could arguably be a more effective method of preventing the NMDA-evoked overflow of glutamate.

It is known that NMDA receptors directly interact with JNK proteins of which, JNK1 and JNK2 isoforms are ubiquitously expressed, while JNK3 is predominantly present in neurons^13,14^. Postsynaptic NMDA receptors strongly facilitate this ‘excitotoxic’ effect by activating JNK proteins with the partial contribution of JIP scaffolding proteins^15^. In support of this, the administration of JNK inhibitor, D-JNKi1, which specifically blocks the interaction of JNK to target proteins carrying the JNK binding domain (JBD) such us JIP-1, is able to prevent NMDA receptor-mediated neuronal death^10,16^.

Presynaptic NMDA receptors are also key players in glutamate neurotransmission^17^ and brain development^18^, as well as in the overflow support of glutamate release^19^ that, in turn, could lead to several pathologies. Very little is known about the role of JNK in the presynaptic compartment, especially in relation to the activity of NMDA receptors, although it has been reported that the contribution of presynaptic NMDA receptors to spontaneous release of glutamate also requires activation of the JNK pathway^20^, and we have recently demonstrated that JNK2 modulates the NMDA-evoked glutamate release, presumably through its interaction with JIP-1^13,21^. In this study we propose Syntaxin-1a (STX1a) as a key JNK2 interactor within this cellular mechanism although further study is required to fully describe it. To elucidate this pathway further, a new Tat-based, cell-penetrating peptide, JGRi1, has been designed to disrupt the interaction between JNK2/STX1a. We show evidence from neurochemical and electrophysiological experiments in which JGRi1 effectively reduced NMDA-driven release of glutamate. Moreover, JGRi1 demonstrated favorable cell-permeability characteristics by easily reaching and diffusing into the brain upon intraperitoneal administration.

Indeed, the JNK2/STX1a interaction has proved to be an interesting new pharmacological target to modulate glutamate overflow, and JGRi1 an attractive compound for further study in models of neuroexcitotoxicity as a neuroprotective agent.

## Results

### NMDA and KCl stimulation modulates presynaptic JNK isoforms and STX1a activity

**Figure 1:**
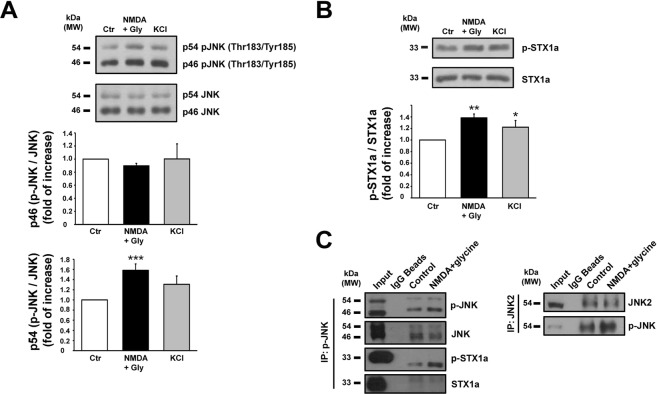
(**A**) Cortical synaptosomes prepared from 3 months wild-type mice have been stimulated with NMDA (100 μM) + glycine (1 μM) for 10 min and with KCl (8 mM) 90 seconds, and then lysated. Mg^2+^-free medium has been used during NMDA stimulus. Quantification of p46-JNK and p54-JNK isoforms phosphorylation has been separately calculated on the cropped WB. Top histogram shows that JNK1 phosphorylation was not affected by stimuli while the lower histogram showed an increased and significant phosphorylation of p54-JNK in correspondence of NMDA stimulus. Means ± s.e.m. n = 6, ***p < 0.001 vs. control; Newman-Keuls’s test. (**B**) The same cortical synaptosomes have been used for the quantification of STX1a phosphorylation on the cropped WB. The phosphorylation of Ser14 of STX1a was increased by both NMDA and KCl stimuli. Means ± s.e.m. n = 6, **p < 0.01 vs. control or *p < 0.05 vs. control; Newman-Keuls’s test. (**C**) IP after stimulus. Cortical synaptosomes underwent NMDA (100 μM) + glycine (1 μM) stimulus for 10 min in Mg^2+^-free medium and together with controls were IP for phosphorylated JNK (left panel) and JNK2 (right panel). Cropped WBs show that p-JNK was augmented by NMDA stimulus and beside the interaction with STX1a, the phosphorylation of STX1a was increased by NMDA respect to control. Non-related IgG magnetic beads and anti IgG secondary antibodies were used as control of the IP quality.

JNK1, JNK2 and JNK3 are alternatively spliced to yield ten proteins that have a molecular weight of either 54 kDa or 46 kDa^22^. Here, we estimated isoform phosphorylation by measuring the relative densitometry of each of the two molecular weight bands. Under NMDA or KCl stimulus, p46-JNK phosphorylation was unchanged while p54-JNK phosphorylation was augmented by NMDA and not by KCl stimulus (1.58 ± 0.12 vs. control, ***p < 0.001) (Fig. 1A). In all experiments, synaptosomes were stimulated in a Mg^2+^-free medium in order to avoid any unwanted blockade of the NMDA receptor^13^.

Through the use of a series of phosphomimetic and phospho-null mutations of STX1a, it has previously been shown that the phosphorylation of STX1a (Ser14 p-STX1a) regulates the N-terminal interaction with Munc18-1^23^, which is a fundamental step for the SNARE (soluble *N*-ethylmaleimide-sensitive factor attachment protein receptor) complex assembly during neurotransmitter release^24,25^. Therefore here we assessed the Ser14 phosphorylation level in stimulated contition and we have found that both NMDA (1.38 ± 0.07 vs. control = 1, **p < 0.01) and KCl (1.22 ± 0.11 vs. control = 1, *p < 0.05) stimuli increased p-STX1a levels in mouse cortical synaptosomes (Fig. 1B).

### JNK interacts with STX1a

To test the interaction between presynaptic STX1a and JNK, mouse cortical synaptosomes stimulated with NMDA were immunoprecipitated (IP) by using a specific p-JNK antibody. The presence of STX1a in both control and stimulated conditions demonstrated that JNK and STX1a interact. It is worth noting that NMDA stimulus increased the level of p-JNK that was accompanied by an augmented pull down of p-STX1a, while the JNK level was unchanged despite the stimulus. These results suggest that the release of glutamate evoked by NMDA is likely to be controlled by the interaction between the phosphorylated JNK and STX1a (Fig. 1C left panel).

IP performed with a specific JNK2 antibody, recognizing the JNK2α2 isoform, confirmed that the NMDA stimulus increased the phosphorylation level of p54-JNK (Fig. 1C right panel). Beads treated with non-related IgG have been used as IP control.

### Docking results for JNK variants and JNK alignment

**Figure 2:**
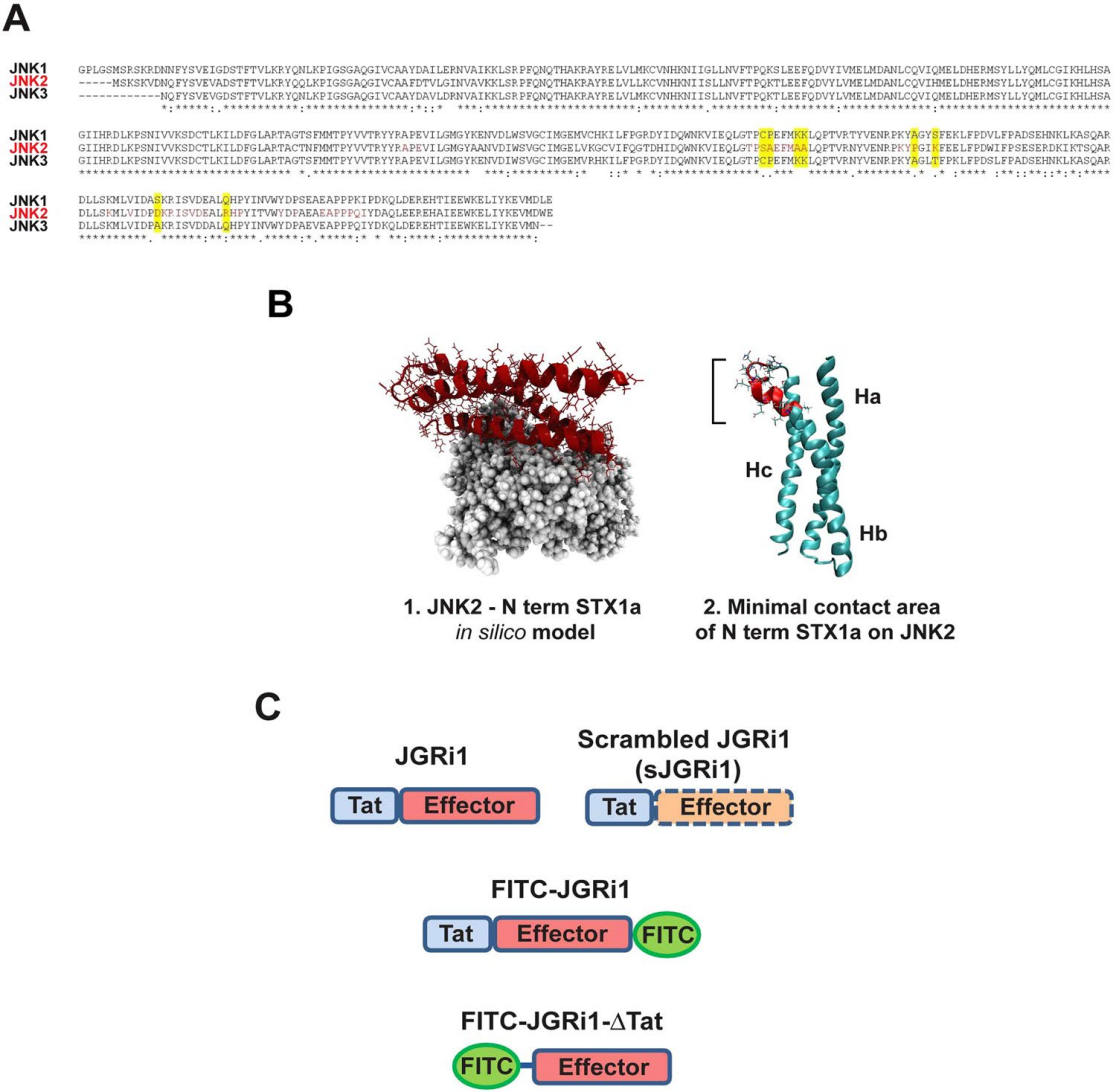
(**A**) The amino acid sequence corresponding to the region of contact between JNK and STX1a has been aligned for all 3 JNK isoforms. It emerged that in that region JNK2 has several specific amino acids while JNK1 and JNK3 are mostly equal. Red box represent the sequence area that is bind by the N-terminal sequence of STX1a. (**B**) 3D structure of the molecular docking resolving the possible interaction between JNK2 (Grey) and the *N*-terminal portion of STX1a (Red) (1). The image originates from a computational analysis that allowed the determination of the ‘minimal contact area’ between the two proteins (2). (**C**) Figurative cartoon that summarizes the peptide forms that have been synthesized and used for the experiments.

Since it has been previously reported that the function of the presynaptic NMDA receptor is reduced in JNK2 knock-out mice^13^ and being evident from our results and published studies that JNK proteins bind to the N-terminal sequence of STX1a^26^, here we performed a sequence alignment of the three known JNK isoforms around the potential interaction area between JNK and STX1a. Interestingly, the analysis showed that the JNK isoforms (−1, −2, −3) present a high sequence homology with the exception of the amino acid sequence of JNK2, in red, that clearly differs from JNK1 and JNK3 (Fig. 2A). Notably, this sequence, calculated by using a protein docking model, corresponded to the most probable interface interaction between JNK2 and the N-terminal sequence of STX1a (105–109 and 90–103 amino acid residues), which it is different from the already known JBD (151–155 and 261–270 amino acid residues)^26^ that correspond with the interaction site of STX1a with all JNK isoforms.

The ‘minimal contact area’ between JNK2 and the N-terminal portion of STX1a was found by running protein docking software Rosetta 3.4^27^ that built an *in silico* JNK2-STX1a complex model (Fig. 2B1) (see computational details). Results from the structural analyses are showing that STX1a binds to JNK2 through the groove formed by two of the three alpha helices that form its structure, namely helix B (Hb) and helix C (Hc), which have therefore been proposed as the most plausible binding site for Syntaxin partners^28^. The ‘minimal contact area’ between the two proteins comprehends an area of 2680 Å highlighted in red (Fig. 2B2).

In order to selectively disrupt JNK2/STX1a interaction, JGRi1, a new 26 amino acid cell permeable peptide, has been designed based on computational data. The 12 residues (GRKKRRQRRRPP) of the HIV-1 Tat protein that confer cell permeability^29^ have been linked to the effector portion (IEQSIEQEEGLNRS) which is part of the N-terminal amino acid sequence of STX1a that correspond to the part of the minimal contact area with JNK2. The scrambled peptide (sJGRi1) only differs from JGRi1 in the effector portion (RISEQLSNIEEGQE) in order to maintain the cell permeability. Several peptide variants have been designed and produced in order to be used in these experiments (Fig. 2C). Besides the computational analysis, biological experiments are required in order to establish that JGRi1 is solely specificity for JNK2/STX1a interaction.

### JGRi1 is able to specifically disrupt JNK2/STX1a interaction

**Figure 3:**
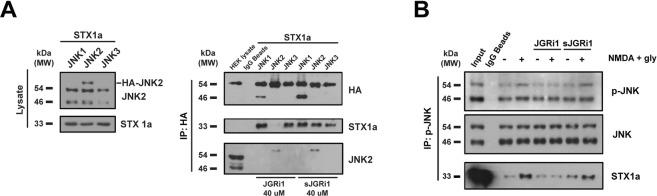
(**A**) HEK-293 cells, transfected with HA-JNK1α2, HA-JNK2α2, or HA-JNK3α2 and STX1a (left blots), were IP for HA and probed for HA, STX1a and JNK2 (right blot) after 40 μM JGRi1 or sJGRi1 treatment (right blots). Cropped WBs are here shown. (**B**) p-JNK IP experiment. JNK phosphorylation was augmented by 10 min NMDA (100 μM) + glycine (1 μM) with respect to not stimulated control, while it was reduced by 30 min pretreatment with JGRi1 (1 μM). The scrambled peptide failed to diminish JNK phosphorylation, demonstrating no effect in reducing the effects of NMDA stimulation. Mg^2+^-free medium has been used during NMDA stimulus. JGRi1 reduced the levels of STX1a, whereas the scrambled version did not. Experiment run in triplicate. Cropped WBs are here shown.

To test the interaction between JNK2 and STX1a we used HEK-293 cells lacking the endogenous expression of STX1a. These cells have been transfected with HA-tagged JNK1, JNK2 or JNK3 together with full length STX1a (non-tagged) plasmids. The presence of the endogenous JNK2 and exogenous STX1a levels was found in all the lanes while HA-JNK2 was present only in the JNK2 lane (Fig. 3A left blots). To HEK-293 cells overexpressing HA-tagged JNK1, JNK2 or JNK3 proteins and STX1a 40 µM of JGRi1 was added or scrambled JGRi1 treatment, and were subsequently immunoprecipitated with HA antibody. Beside the presence of all HA-JNK proteins, as expected, the treatment with JGRi1 prevented the interaction between STX1a and HA-JNK2, but not with HA-JNK1 and HA-JNK3. On the other hand, sJGRi1 did not show any JNK/STX1a disrupting effect (Fig. 3A right blots).

### JGRi1 disrupts JNK/STX1a interaction in mouse cortical synaptosomes

The JNK/STX1a interaction was also tested in cortical synaptosomes that were previously treated and immunoprecipitated for p-JNK. NMDA stimulus increased the phosphorylation of both p46 and p54 isoforms of JNK compared to the unstimulated control sample, accompanied by a strong augmented interaction between p-JNK and STX1a. Pretreatment with JGRi1 alone did not show any effect on the basal level of p-JNK while, interestingly, prevented the phosphorylation of both p46 and p54 isoforms of JNK following NMDA. As expected, pretreatment with JGRi1 reduced the interaction of p-JNK with STX1a only during NMDA stimulus (Fig. 3B). Conversely, sJGRi1 did not prevent the NMDA-mediated p-JNK increase, nor the disruption of the JNK/STX1a interaction.

### JGRi1 doesn’t impair JNK phosphorylation while affects STX1a activation

**Figure 4:**
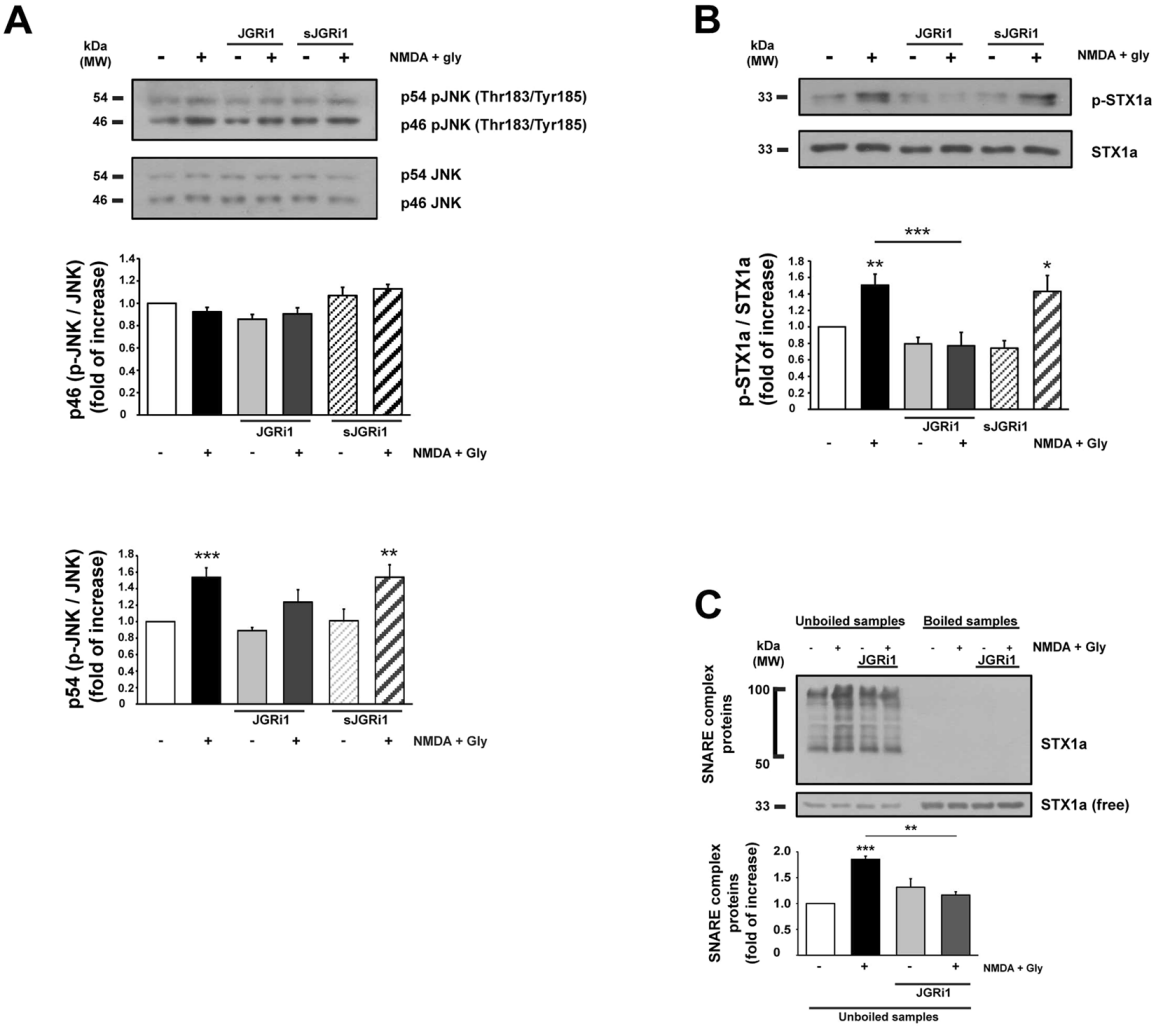
(**A**) p46-JNK and p54-JNK level measurement. p46-JNK was not affected by treatments while p54-JNK was increased after 10 min NMDA (100 μM) + glycine (1 μM) alone or pre-treated with JGRi1 (1 μM) or sJGRi1. JGRi1 (1 μM) or sJGRi1 (1 μM) alone did not affect JNK phosphorylation state. Means ± s.e.m. n = 7, ***p < 0.001 NMDA stimulus vs. control, **p < 0.01 NMDA + sJGRi1 vs. control; Newman-Keuls’s test. Cropped WBs are here shown. (**B**) NMDA stimulus increased STX1a phosphorylation that was instead prevented by pretreatment with JGRi1 (1 μM), but not by sJGRi1 (1 μM). Means ± s.e.m. n = 7, *p < 0.01 NMDA stimulus vs. control, ***p < 0.001 JGRi1 alone vs NMDA, ***p < 0.001 NMDA + JGRi1 vs NMDA, *p < 0.05 NMDA + sJGRi1 alone vs. control; Newman-Keuls’s test. Cropped WBs are here shown. (**C**) Where indicated JGRi1 (1 μM) was applied to cortical synaptosomes 30 min before 10 min NMDA (100 μM) + glycine (1 μM) stimulus. SNARE complex formation was assessed by probing STX1a in unboiled samples comparing STX1a lanes smearing (50 to 100 kDa) with free STX1a in a cropped WB. NMDA increased SNARE complex formation while JGRi1 prevented it. Means ± s.e.m. n = 4, ***p < 0.001 NMDA stimulus vs. control, **p < 0.01 JGRi1 alone vs NMDA; Newman-Keuls’s test.

JNK isoforms activation has been evaluated in cortical synaptosomes that underwent NMDA stimulation. An increased level of p54-JNK phosphorylation was detected after NMDA treatment alone (1.54 ± 0.12 vs. control = 1, ***p < 0.001), in contrast to pretreatment with JGRi1 decreased it (1.24 ± 0.15 vs. control = 1). sJGRi1 was not able to prevent NMDA-evoked p54-JNK phosphorylation (1.54 ± 0.15 vs. control = 1, **p < 0.01). In basal conditions, p-JNK2 levels were not changed by pretreatment with either JGRi1 or sJGRi1, and p46-JNK phosphorylation level was not affected in any condition studied (Fig. 4A).

Conversely, STX1a phosphorylation was increased by NMDA treatment (1.51 ± 0.13 vs. control = 1, **p < 0.01), while JGRi1 pretreatment abolished NMDA effect and sJGRi1 was again ineffective (1.43 ± 0.19 vs. control = 1, *p < 0.05). Both JGRi1 and sJGRi1 applied alone did not evoke STX1a phosphorylation (Fig. 4B).

### JGRi1 prevents the formation of SNARE complex

Biochemical evidence of the inhibitory effect of JGRi1 on the release of glutamate was measured by performing a SNARE complex formation assay, where SNARE complex assembly is a crucial step for the release of neurotransmitters. In fact, it is possible to measure the formation of the SNARE complex by probing unboiled samples for STX1a from cortical synaptosomes^26,30^. As expected, NMDA stimulus increased the formation of the SNARE complex (1.85 ± 0.06 vs. control = 1, ***p < 0.001) while JGRi1 was able to prevent it (1.16 ± 0.06 vs. NMDA, **p < 0.01) (Fig. 4C).

### JGRi1 reduces presynaptic NMDA-evoked release of neurotransmitter

**Figure 5:**
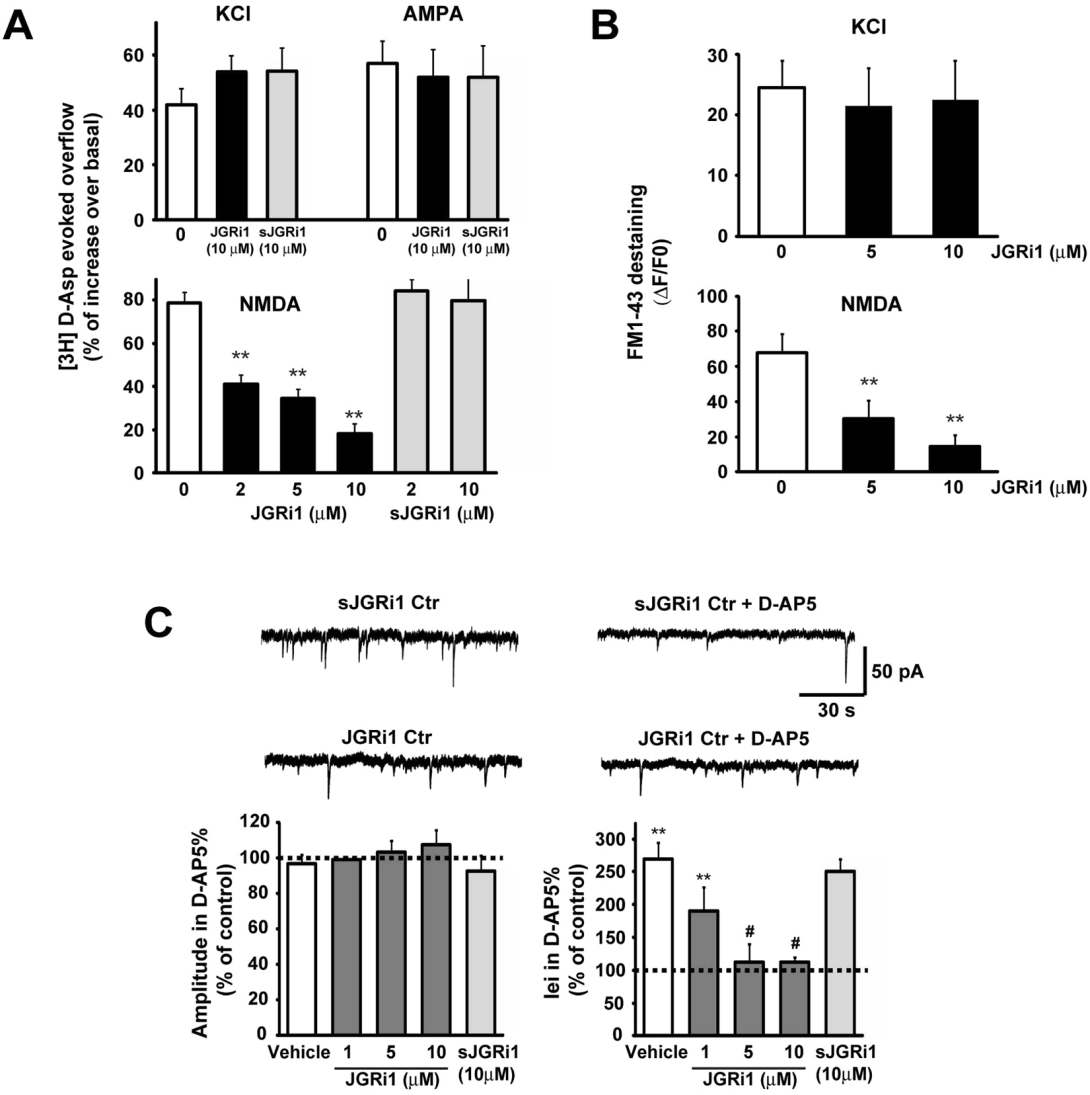
(**A**) Wild-type mice cortical synaptosomes preloaded with radioactive tracer were incubated with several stimuli in absence or presence of JGRi1 or sJGRi1, as indicated. The experiment demonstrated that, among the applied stimuli, JGRi1 is able to specifically inhibit only the NMDA-evoked glutamate release in a dose dependent manner, while scramble peptide is not. Means ± s.e.m. n = 3 experiments run in triplicate (three superfusion chambers for each experimental condition), **p < 0.01 vs. basal release; Newman-Keuls’s test. (**B**) Exocytosis evoked by KCl (8 mM) or NMDA (100 μM) + glycine (1 μM) stimuli has been evaluated in cortical synaptosomes as a measure of FM1-43 destaining in time-lapse image experiments. Pretreatment with JGRi1 at different concentrations given 30 min before the registration specifically reduced NMDA-evoked exocytosis. Average percentage of fluorescence loss has been reported in the histograms. Means ± s.e.m. n = 5, **p < 0.01 vs. control; Newman-Keuls’s test. In each experiment three coverslips for each experimental group were analysed. (**C**) Histograms show the median values expressed as percentage versus control (100%) of mEPSC amplitude (left) and iei (right) recorded in vehicle, JGRi1 (1-10 μM) or scrambled condition (sJGRi1 10 μM), in response to D-AP5. mEPSCs were detected in the presence of extracellular tetrodotoxin (TTX, 1 μM), picrotoxin (PTX, 100 μM) and MK-801 (10 μM) in the patch-pipette (in order to block NMDA receptors in the cell recorded). Means ± s.e.m. n = 8, **p < 0.01 vs. ctrl, #p < 0.05 vs. JGRi1 (1 μM), *t*-test).

The evoked tritium aspartic acid ([^3^H]D-Asp) overflow from cortical synaptosomes was evaluated in different conditions. Pretreatment with several doses of JGRi1 reduced the NMDA-evoked release of [^3^H]D-Asp in a dose dependent manner (2 µM = 41% ± 5 vs. 0 µM, **p < 0.01; 5 µM = 34% ± 4 vs. 0 µM, **p < 0.01; 10 µM = 18% ± 4 vs. 0 µM, **p < 0.01) while KCl and AMPA-evoked release of [^3^H]D-Asp was not affected even with the highest dose of JGRi1. Synaptosomes pretreatment with sJGRi1 did not affect the NMDA-evoked release of [^3^H]D-Asp at any dose tried (2 µM = 84% ± 5; 10 µM = 79% ± 10) (Fig. 5A).

In parallel, synaptic vesicular release has been tested in cortical synaptosomal preparations as previously described, by means of FM1-43 run-down^13^. Interestingly, NMDA-induced release of fluorescent dye (68% ± 10) whereas pretreatment with JGRi1 reduced the evoked vesicle release in a dose-dependent manner (5 µM = 27% ± 8 vs. 0 µM, **p < 0.01; 10 µM = 18% ± 4 vs. 0 µM, **p < 0.01). KCl-evoked vesicle release was not affected, probably because this stimulus is not correlated with the activation of JNK as already reported^13^. Therefore, these data show that JGRi1-mediated vesicle release inhibition is specific for NMDA receptors (Fig. 5B).

### JGRi1 blocks spontaneous presynaptic NMDA currents

The effect of JGRi1 on NMDA receptor-dependent presynaptic glutamate release was evaluated by measuring miniature excitatory postsynaptic currents (mEPSCs), according to a protocol previously described^13^. In basal conditions, mEPSCs are tiny and rapid inward currents deriving from the vesicular release of neurotransmitter that represents the presynaptic terminal activity reaching the patched postsynaptic neuron. The higher the frequency of mEPSCs, the stronger the connectivity of the cell tested. Inter-event interval (iei) can be considered an indirect measure of mEPSCs frequency. Consequently, the increase of iei indicates a decrease in NMDA receptors firing, confirming that NMDA receptors clearly contribute to the depolarization of the presynaptic terminal.

Application of D-AP5 in the presence of extracellular tetrodotoxin (TTX, 1 μM), picrotoxin (PTX, 100 μM), and MK-801 (10 μM) in the patch-pipette (in order to block NMDA receptors in the cell we were recording from^31^) induced an increase of iei (**p < 0.01, t-test, vehicle vs. D-AP5, n = 8; Fig. 5C), whilst the amplitude of the same events was unaffected (*p > 0.05 Student t-test, vehicle vs. D-AP5, n = 8; Fig. 3C). In slices pre-incubated for 30 min, we found that infusion of D-AP5 did not lead to significant changes of inter-event intervals when JGRi1 was applied at 5 μM and 10 μM (*p > 0.05, t-test, JGRi1 5 μM vs. D-AP5, n = 7; *p > 0.05, t-test, JGRi1 10 μM vs. D-AP5, n = 9; Fig. 5C), indicating that presynaptic NMDA receptors inhibition has been effectively accomplished by JGRi1, since the pharmacological inhibition by D-AP5 did not further reduce the iei. On the other hand, no changes were observed in the amplitude of mEPSCs under the same conditions, suggesting that JGRi1 does not influence the biochemistry of glutamate release. Overall these electrophysiological results indicate that JGRi1 reduces glutamate release mediated by presynaptic NMDA receptors.

### JGRi1 diffuses in the brain following *in-vivo* administrations

**Figure 6:**
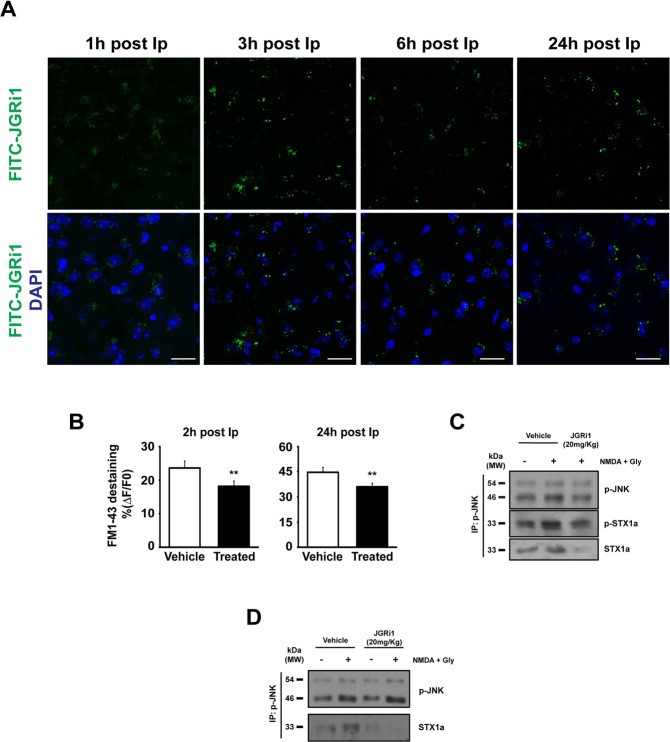
(**A**) FITC-JGRi1 (20 mg/kg) was intraperitoneally administered and its brain distribution was detected by confocal microscopy. Cortical tissue has been examined at several time points and the FITC fluorescence was detected until 24 h after injection. Scale bar = 15 μm. (**B**) JGRi1 (20 mg/kg) was intraperitoneally administered and after 2 h or 24 h the cortex was used to prepare synaptosomes and to test the exocytosis evoked by NMDA (100 μM) + glycine (1 μM) stimulus measured as FM1-43 destaining in a time-lapse image experiments. The injected JGRi1 was able to reduce NMDA-evoked exocytosis in both time points at a comparable strength. Means ± s.e.m. n = 3, **p < 0.01 vs. control, *t*-test. (**C**) Mice were treated intraperitoneally with 1 injection of vehicle or JGRi1 (20 mg/kg) and after 24 h the cortex was used to prepare synaptosomes that were stimulated by NMDA (100 μM) + glycine (1 μM) as indicated. Synaptosomes were then IP for p-JNK and probed for p-JNK. NMDA treatment increased the phosphorylation of JNK while JGRi1 decreased the level of both pSTX1a and STX1a, showing the efficacy of the disruption activity of the peptide. NMDA stimulus alone, as expected, was able to increase the interaction between p-JNK and p-STX1a. Cropped WBs are here shown. (**D**) Mice were treated intraperitoneally with 1 injection per day of vehicle or JGRi1 (20 mg/kg) and after 7 days the cortex was used to prepare synaptosomes that were stimulated by NMDA (100 μM) + glycine (1 μM), as indicated. Synaptosomes were then IP for p-JNK and probed for p-JNK and STX1a. NMDA alone increased the interaction between p-JNK and STX1a while JGRi1 was able to reduce their interaction in both control and NMDA conditions. Cropped WBs are here shown.

Peptides containing HIV-Tat_(47–57)_ are able to cross the blood brain barrier (BBB) within 1 h after injection^32,33^. As expected, FITC-JGRi1 was detectable in the cortex (Fig. 6A) from 1 h till 24 h post intraperitoneal (Ip) injection, as well as in hippocampus 24 h post Ip injection (Supplementary Fig. S1B). The brain diffusion of FITC-JGRi1 has been also monitored by analyzing confocal image of a representative large cortical-hippocampal area of the brain (Fig. S2). The FITC-JGRi1 was still present after 24 post injection in whole slice and it is especially well visible in the hippocampal area (see arrowed dots in the gray image). The Fluorescein (FITC) tag linked at the *C*-term of the Tat-peptide (Fig. 2C) allowed us hypothesize that the whole peptide is still intact when it reaches the brain. In fact, a variant version lacking the Tat portion (FITC-JGRi1-ΔTat, Fig. 2C) was not able to reach cortical tissues (Supplementary Fig. S1A).

### JGRi1 shows brain activity after *in-vivo* Ip injection

Mice injected with a single dose of JGRi1 or vehicle were used to prepare cortical synaptosomes after 2 or 24 h post Ip injection. NMDA-evoked vesicle release was measured and JGRi1 treated mice showed a lower vesicle release with respect to vehicle alone (2 h: −23% ± 7% JGRi1 vs. vehicle; 24 h: −26% 4 JGRi1 vs. vehicle) (**p < 0.01) (Fig. 6B), showing that a single injection of the peptide, up to 24 h, still induces an efficient inhibition of NMDA receptor activity.

In order to test the peptide ability to reach its molecular target and to be effective *in-vivo*, the disruption activity upon p-JNK/STX1a was tested preparing synaptosomes from mice sacrificed 24 h after a single Ip dose of JGRi1. The IP for p-JNK showed that NMDA treatment induced an increased the level of pJNK in vehicle animal, interacting more strongly with STX1a with respect to basal conditions. The p-STX1a level was also increased by the NMDA treatment, as expected. Coherently, JGRi1 efficiently inhibited NMDA activation of p-JNK which was almost restored back to basal condition; moreover JGRi1 disrupted JNK/STX1a interaction since the levels of STX1a, and consequently its phosphorylation, were drastically decreased (Fig. 6C).

In addition, to study the molecular target of the peptide in a longer *in-vivo* paradigm, a single dose of JGRi1 was administered every day for 7 days and used to prepare cortical synaptosomes that were subsequently treated with NMDA, as indicated, and IP for p-JNK. Again, the level of pJNK was increased by NMDA stimulus compared to basal condition in both animals treated with vehicle and JGRi1, but the interaction with STX1a was completely abolished in the sole JGRi1 treated animals. This result once again demonstrated the efficacy of the peptide in disrupting JNK/STX1a interaction (Fig. 6D).

## Discussion

NMDA receptors are known to support ‘excitotoxicity’ in several medical conditions including cerebral ischemia, epilepsy and neurodegenerative disorders, wherein glutamate overflow is the major etiopathological culprit^11,12,34^. The efforts of pharmaceutical research to find an efficient NMDA inhibitor to contrast ‘excitotoxicity’ have been unsuccessful in the last decade^5^. One of the hypotheses for the failure of these molecules in clinical trials may be the inappropriate pharmacological action of these molecules that indiscriminately antagonize the NMDA receptor, blocking the flow of ions through the channel, thereby compromising the normal neurotransmission in certain areas of the brain, and consequently leading to harmful adverse effects^35^. A more accurate knowledge of the NMDA receptors intracellular signaling could lead to the identification of new therapeutic targets that might be more specific and therefore safer.

It is widely recognized that postsynaptic NMDA receptors drive the apoptotic signal through intracellular activation of JNK proteins mediating ‘excitotoxicity’ both in the brain^15^ and spinal cord^36^. It has been recently shown that presynaptic JNK controls the NMDA-evoked release of glutamate, becoming an interesting intracellular target in ‘excitotoxic’ conditions, thereby worthy of further study.

In this paper we introduce a new molecule that has been specifically designed to inhibit the presynaptic NMDA receptor by blocking an intracellular pathway that has been discovered here involves JNK proteins^13^.

We have previously demonstrated that the overflow of glutamate evoked by NMDA presynaptic receptors is specifically mediated by JNK2 because JNK2 knock-out mice showed a drastic inhibition of glutamate release upon NMDA stimulus^13^ in biochemical, neurochemical and electrophysiological experiments.

Here, we additionally show that the activation of the presynaptic NMDA receptor induces a strong JNK-STX1a interaction, which in turn controls the release of glutamate. STX1a is a specific presynaptic protein, part of the SNARE complex, that regulates the presynaptic neurotransmitter release^37^, including glutamate^38^. Although its physiological activity is complex and not totally defined, STX1a phosphorylation at Ser14, potentially driven by Casein Kinase II, is a crucial step for the formation of the SNARE complex. Interestingly, Ser14-phosphorylated STX1a has also been found to be correlated with schizophrenia, showing a 25% reduction, corresponding with a decrease in protein kinase CK2^39^. The phosphorylation of STX1a seems to be triggered by the raising of the intracellular concentration of Ca^2+^ in charge of presynaptic membrane depolarization, like NMDA or KCl stimulus^40,41^.

It was previously found that both STX1a and STX2 (151–155 and 261–270 amino acid residues) contain a JNK binding domain (JBD) and that JNK1 co-immunoprecipitates with both STX1a and 2 while JNK2 and 3 interact preferentially with STX2^26^.

In addition to published data, our computational analysis predicted that JNK2 interacts with STX1a with an higher probability respect to JNK1 and 3 through an amino acid sequence (105–109 and 90–103 amino acid residues) different from the classical JBD. Although this data is coming exclusively from computational experiments we think that is interesting for future investigation, to prove whether this putative interaction between JNK2 and STX1a would be more specific than the interaction of other JNK isoforms with STX1a.

The identification of a putative amino acid sequence of STX1a that interacts with JNK2 has been exploited to produce a cell permeable peptide (CPP) containing the Tat amino acid sequence that confers cell permeability^29^.

CPPs are interesting pharmacological tools that are currently widely used in research in order to specifically interfere with cellular functions^10,42^ and some of them are following the pharmaceutical clinical phases for human pathologies^43,44^. In line with this, our new CPP (JGRi1), but not the scrambled sequence (sJGRi1), is able to efficiently interrupt JNK/STX1a protein-protein interaction and, in turn, to reduce the NMDA-evoked release of glutamate, without affecting the basal glutamatergic transmission.

We demonstrated here that JGRi1 reduced JNK/STX1a interaction causing a reduction of STX1a phosphorylation at Ser14, ultimately preventing the SNARE complex formation specifically under ‘excitotoxic’ stimuli.

JGRi1 was designed exactly on that JNK2/STX1a specific amino acid region and, coherently, it efficiently disrupted the JNK2/STX1a interaction, but not that of JNK1 or 3/STX1a in overexpressing experiments performed in HEK-293 cells. Other experiments are necessary to test the JNK isoform specificity. In general, in our experiments we showed that JGRi1 is able to reduce the presynaptic activation of JNK trough NMDA presynaptic receptors. Additionally, JGRi1 was also able to reach its specific molecular target in *in-vivo* experiments. By targeting JNK2/STX1a interaction we propose that JGRi1 could be specific to disrupt JNK2/STX1a interaction compared to JNK1 and JNK3 with STX1a but still other experiments are under development to prove this specificity.

To date, neuroprotection from glutamate toxicity remains a fundamental problem in clinical practice as no anti-excitotoxic drug has been proposed in the field of preventative therapy.

The results present here show for the first time the usa of a molecule targeting JNK2/STX1a interaction, therefore a presynaptic protein interaction, is able to modulate presynaptic signaling of NMDA receptors and possibly to prevent the overflow of glutamate.

Conclusively, the data collected in this paper demonstrate that JNK2/STX1a interaction is a potential intracellular presynaptic mechanism that modulates the NMDA-evoked glutamate release. JGRi1, as a new molecule, can represent a prototype tool to be tested in various rodent models of neuroexcitotoxicy, including stroke, epilepsy and chronic neurodegenerative diseases.

## Material and Methods

### Animals

3 months old C57BL/6 mice were anesthetized with 5% isoflurane and killed by decapitation, in accordance with the guidelines established by the European Communities Council (Directive 2010/63/EU of 22 September 2010) and accepted by the Italian Ministry of Health and approved by the Ethical Committee on animal experiments of EBRI “Rita Levi-Montalcini” Foundation (Rome, Italy). Brains were rapidly dissected out on ice.

### Synaptosomal preparation

Synaptosomes are a widely diffused neuronal preparation that allows, by exogenous stimulation, the study of synaptic protein function, glutamate release, and synaptic vesicle trafficking^45–47^. Synaptosomes prepared as previously described^47^.

Briefly, C57BL/6 mice cortices were homogenized in 0.32 M Sucrose and 0.01 M Tris (pH 7.4) and centrifuged 5 min at 1000 × g to remove nuclei and debris. The synaptosomal fraction was isolated a discontinuous Percoll gradient between 10 and 20% layers and the pellet resuspended in a physiological solution (Saline Buffer pH 7.2–7.4): NaCl 140 mM, KCl 3 mM, MgSO_4_ 1.2 mM, CaCl_2_ 1.2 mM, NaHPO_4_ 1.2 mM, NaHCO_3_ 5 mM, HEPES 10 mM, Glucose 10 mM.

### Synaptosomal stimulation in biochemical studies

Stimulation of synaptosomes was performed in batch as previously reported^13^. Synaptosomes were stimulated with either NMDA (100 μM) + glycine (1 μM) or KCl (8 mM) respectively for 10 min at 37 °C or for 90 seconds after which warm medium containing inhibitors, where needed, was added. JGRi1 or sJGRi1 (1 μM) were added, where indicated, for 30 min before NMDA stimulus and till the end.

In order to avoid the physiological NMDA receptor Mg^+2^ block, the saline buffer was replaced, 10 min before the NMDA stimulation, with a Mg^2+^-free saline buffer as already reported^13^. Synaptosomes were then centrifuged and the final pellets were lysed in Lysis Buffer solution (LB) and protein prepared for western blot.

### Radioactive glutamate release experiments

Cortical synaptosomes were loaded with [^3^H]D-aspartate ([^3^H]D-Asp final concentration 50 nM) and JGRi1 was added at different concentrations for 30 min. Synaptosomes were superfused at 37 °C layered in a Superfusion System (Ugo Basile, Comerio, Varese, Italy) for 48 min as previously reported^13^.

Indicated stimuli were applied and fractions collected and superfused synaptosomes were counted for radioactivity. The amount of radioactivity released into each superfusate fraction was expressed as a percentage of the total synaptosomal tritium content at the start of the fraction collected (fractional efflux). This method of superfusion avoids any possible cross-reactivity of the exocytotic neurotransmitter release.

### HEK-293 cell cultures and transfection

HEK-293 cells were seeded and maintained at 37 °C under 5% CO_2_ in complete DMEM (LONZA Group LTD, Switzerland) with 4.5 g/l glucose serum.

Co-transfections of pCMV5-STX1a (1 µg) plasmid and one of HA-JNK1α2, HA-JNK2α2, or HA-JNK3α2, as indicated, were performed using Lipofectamine® 2000 (Invitrogen, USA). The following day, JGRi1 (40 µM) was added for 1 h 30′. Cells were harvested in Lysis Buffer solution and 350 µg of protein from each sample was immunoprecipitated with mouse anti-HA 1:1000 (#H3663,Sigma-Aldrich, USA).

### Western blot

Western blot analyses were performed as previously reported^13^ using the following primary antibodies: rabbit anti-STX1a 1:1000 (#110302, Synaptic System, Germany), rabbit anti-p-STX1a 1:1000 (#ab63571, AbCam, USA), rabbit anti-JNK 1:1000 (#9252, Cell Signaling, USA), rabbit anti-p-JNK 1:1000 (#9251, Cell Signaling, USA), rabbit anti-JNK2 1:1000 (#ab76125, AbCam, USA) and mouse anti-SNAP25 1:1000 (#836303, Biolegend, USA).

Non-related Light Chain specific anti-IgG specific secondary antibodies were used for IP blotting (#211-002-171, Jackson Lab, USA).

### Computational details

The structures of JNK2 and of the *N*-terminal domain of STX1a were obtained from Protein Data Bank PDB ids 3NPC^48^ and 1BR0^28^ respectively. Docking simulations were performed using the Rosetta 3.4 software^27^. One hundred thousand docking decoys were generated and ranked according the total score, which took into account contributions of several chemical/physical characteristics. Sequence alignment of the JNK1, JNK2 and JNK3 has been performed with Clustal Omega^49^ using default options. The JNK2 domain binding to STX1a upon complex formation showed several mutations with respect to analogue domains in JNK1 and JNK3, while the three proteins on the whole displayed a high sequence identity.

### Peptide synthesis

The peptides were prepared by microwave-assisted solid phase synthesis^50^ based on 9-Fluorenylmethyloxycarbonyl (Fmoc) chemistry on pre-loaded Wang resin (0.4 meq/g substitution), choosing a fivefold molar excess of 0.2 M Fmoc-protected amino acids dissolved in N-methyl pyrrolidinone, and using HOBT/HBTU/DIEA (5: 5: 10 eq) as activators.

Some peptides were then N-terminal or C-terminal labeled with FITC to be then studied with confocal images.

### Immunoprecipitation experiments

350 µg of protein extract in Lysis Buffer containing protease and phosphatase inhibitors were immunoprecipitated using non-related IgG magnetic beads (New England BioLabs, USA) and rabbit anti-p-JNK antibody 1: 200, (#9251, Cell Signaling, USA) in every sample excluding total Lisate.

Beads were then washed and the immunoprecipitated complexes were prepared for immunoblot analysis.

### Electrophysiological experiments

Two months old C57BL/6 mice were anesthetized with halothane and killed by decapitation. The brain was rapidly removed from the skull and 250 μm combined entorhinal-hippocampal slices were obtained as previously described^13^. Whole cell patch clamp recordings were performed on layer II pyramidal neurons of the entorhinal cortex (EC) following a previous report^13^.

### Measurements of synaptic release

Synaptogreen (SIGMA-ALDRICH, Italy) (FM1-43) was used to measure synaptic release as previously described^47^. Briefly, cortical synaptosomes from non-treated mice or after 2 or 24 h Ip injection were loaded with FM1-43 (50 μM) and synaptosomes were then seeded on coverslips. Registration of fluorescent dye release was performed on a time-lapse system. Video recordings were performed for 6 (8 mM KCl) or 30 min (NMDA 100 μM + glycine 1 μM) by taking 14 bit images every 2 s. Quantification of FM1-43 responses was accomplished by calculating the average percentage of fluorescence loss.

### SNARE complex assay

The modulation of the complex assembly upon NMDA (100 µM) + glycine (1 μM) or JGRi1 (1 µM) stimulation has been tested on cortical synaptosomes and evaluated via Western blot, comparing unboiled with boiled (5 min, 95 °C) samples. The immunoreactive bands identified by rabbit anti-STX1a 1:1000 (#110302, Synaptic System, Germany) at approximately 50–100 KDa were quantified as an indicator of the SNARE complex assembly upon treatment.

### IP administration

Systemic intraperitoneal injections were performed to administer JGRi1 peptide variants for subsequent analysis, according to our experimental purposes. FITC-JGRi1 (20 mg/kg) was administered intraperitoneally to 3 months old wild-type mice in order to evaluate its brain distribution.

### Confocal images

3 months old wild-type mice were deeply anesthetized with tribromoethanol (Avertin®) and perfused through the aorta with ice-cold 4% paraformaldehyde. Brains were post-fixed for at least 4 h at 4 °C and equilibrated with 30% sucrose overnight. Thirty micrometer-thick sections of perfused brains were permeabilized in PBS with Triton-X 0.25% (TPBS) and pre-incubated with 10% normal donkey serum solution for 1 h at room temperature containing DAPI 0.01 mg/ml.

Sections were mounted on a coverslip and examined under a confocal laser scanning microscope (Leica SP5, Leica Microsystems, Wetzlar, Germany), equipped with 4 laser lines and a transmitted light detector for differential interference contrast (DIC; Nomarski) imaging. Confocal acquisition setting was kept identical among the slides and throughout the whole acquisition.

The landscape image was constructed by appropriately overlapping consecutive frames collected from brain slices fixed from injected mice.

### Statistical analysis

For animal experiments: ANOVA test was used together with Newman-Keuls multiple-comparison test or alternatively student’s two-tailed t-test when needed using GraphPad PRISM 4 (GraphPad software, USA).

## Supplementary information

Supplementary Figs
