# Supplementary material for: The selective disruption of presynaptic JNK2/STX1a interaction reduces NMDA receptor-dependent glutamate release
Source: Sci Rep. 2019 May 9;9:7146. doi: 10.1038/s41598-019-43709-2 (PMC6509125; doi:10.1038/s41598-019-43709-2)
Supplement: Supplementary file 1 — Supplementary Figs [file 41598_2019_43709_MOESM1_ESM.pdf]

The selective disruption of presynaptic JNK2/STX1a interaction reduces NMDA receptor-dependent glutamate release

Serena Marcelli[1,#], Filomena Iannuzzi[1,#], Elena Ficulle[2,#], Dalila Mango[3], Stefano Pieraccini[4,5], Sara Pellegrino[6], Massimo Corbo[2], Maurizio Sironi[4,5], Anna Pittaluga[7,8], Robert Nisticò[3,9], Marco Feligioni[1,2*]

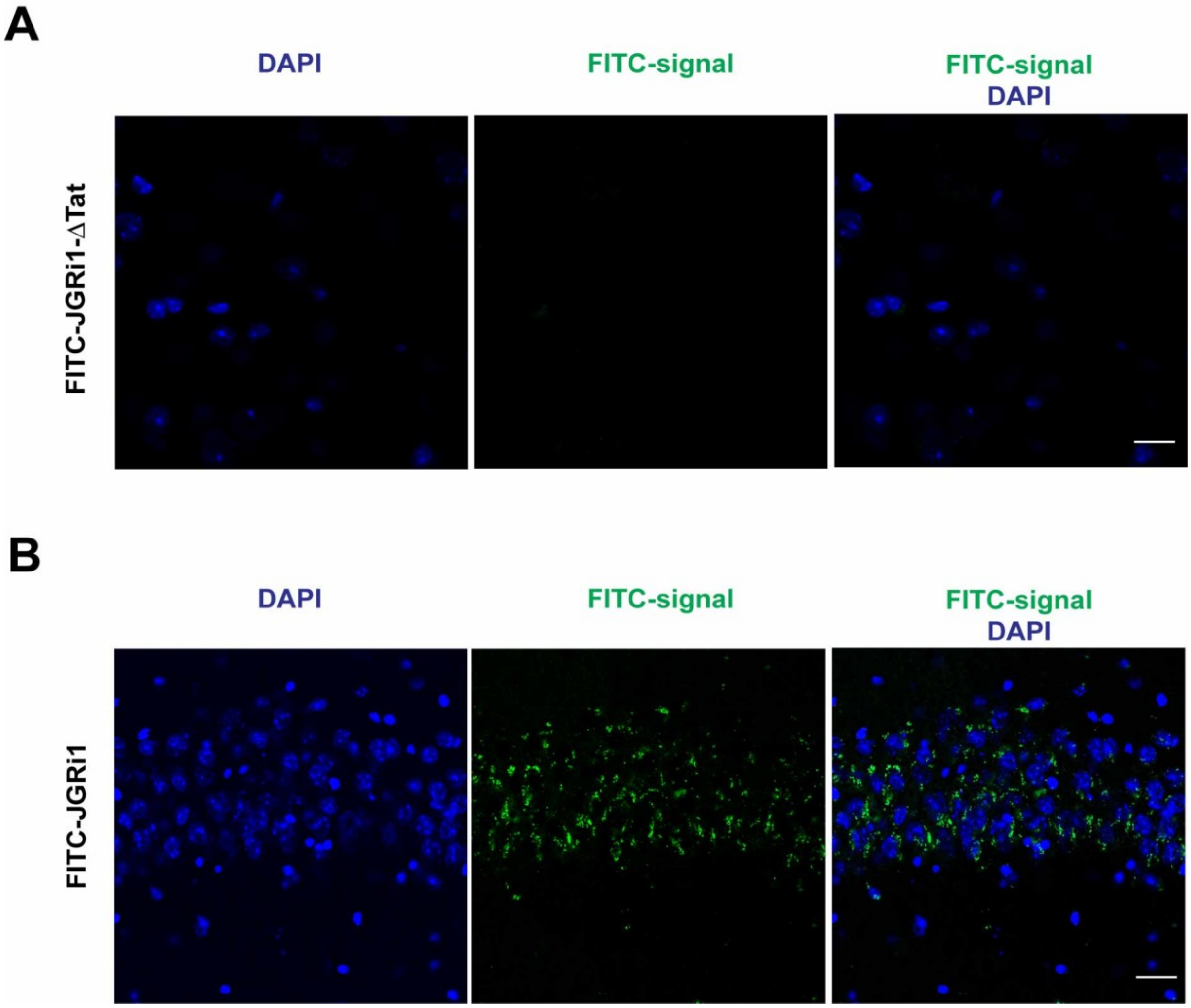

Figure S1.

A) FITC-JGRi1-ΔTat (20mg/kg) was intraperitoneally administered and its brain distribution detected by confocal microscopy. Cortical tissue has been examined 3 h after injection and no FITC fluorescence was detected. Scale bar = 15 µm.

B) FITC-JGRi1 (20mg/kg) was intraperitoneally administered and its brain distribution detected by confocal microscopy. Hippocampal tissue has been examined at several time points and the FITC fluorescence was detected until 24 h after injection. Scale bar = 15 µm.

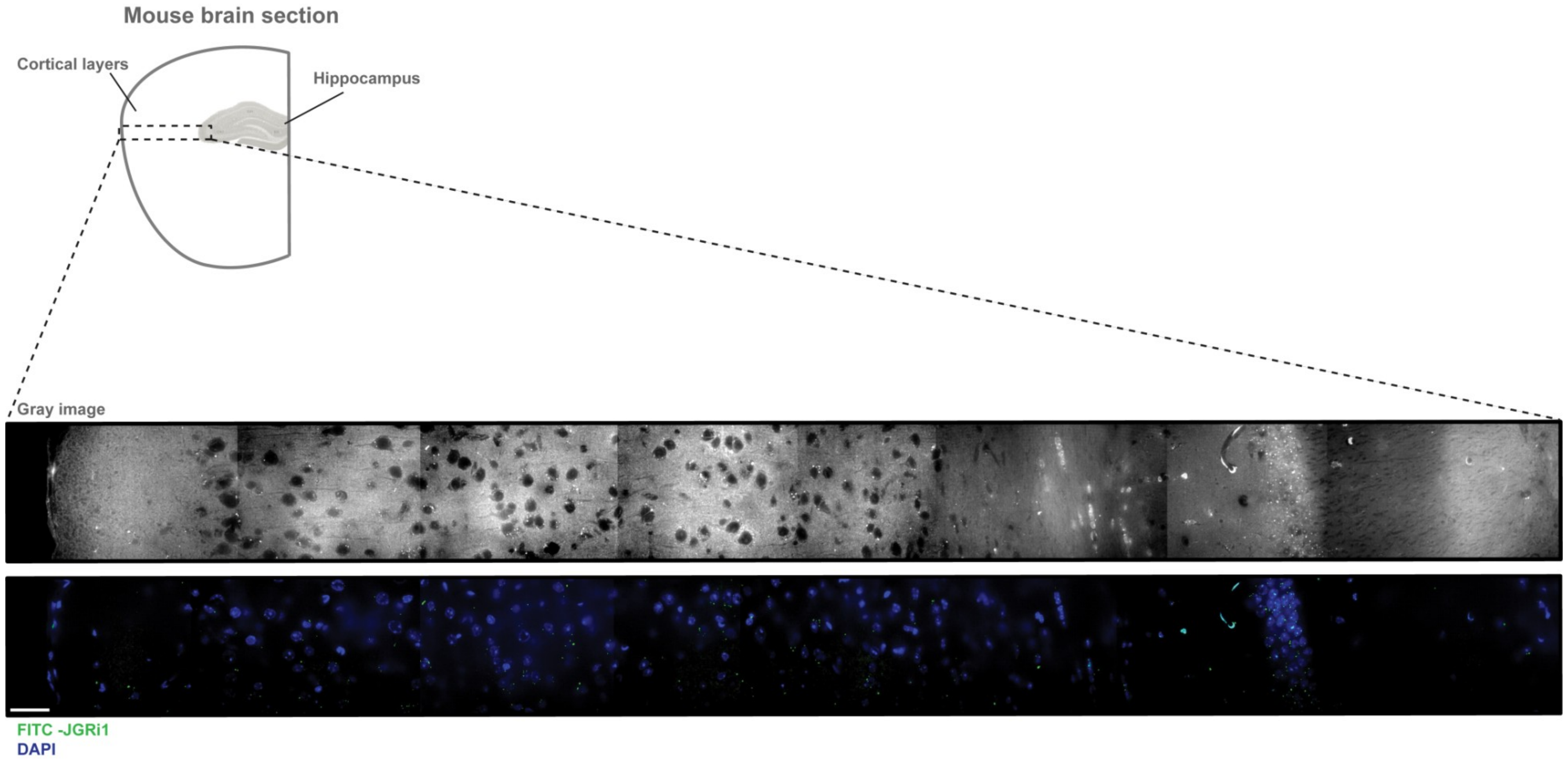
Mouse brain section
Cortical layers
Hippocampus
Gray image
FITC -JGRi1
DAPI

Figure S2.

FITC-JGRi1 (20mg/kg) was intraperitoneally administered and its brain distribution detected by confocal microscopy. Cortical tissue has been examined 24 h after injection. The region of interest has been indicated on the representative section drawn in the cartoon. The gray image is a collage of several field of observation, at 60x magnification, of a representative area of the brain from cortical-to-hippocampal sites. The FITC-JGRI1/DAPI image of the same area is here showed. Scale bar = 10 µm.
